# Epigenetic-related transcriptional reprogramming elucidated by identification and validation of a novel reference gene combination for RT-qPCR studies in porcine oocytes of contrasting quality

**DOI:** 10.1007/s11033-024-09319-6

**Published:** 2024-02-27

**Authors:** Linda Marijke Haug, Robert C. Wilson, Anne Hege Alm-Kristiansen

**Affiliations:** CRESCO, Centre for Embryology and Healthy Development, Department of Biotechnology, Inland Norway University of Applied Sciences, Hamar, Norway

**Keywords:** RT-qPCR normalisation, *PFKP*, Gilts, Sows, Histone modification

## Abstract

**Background:**

Reliable RT-qPCR results are dependent on appropriate normalisation. Oocyte maturation studies can be challenging in this respect, as the stage of development can distinctively affect reference gene transcript abundance. The aim of this study was to validate the use of reference genes in oocyte in vitro maturation RT-qPCR studies, and thereafter, examine the abundance of transcripts supporting histone modification during oocyte and early embryo development in oocytes of contrasting quality.

**Methods and results:**

Total RNA from oocytes from prepubertal gilts and sows was extracted either directly succeeding follicle aspiration or after 44 h in vitro maturation, followed by RT-qPCR. The stability of *YWHAG*, *HPRT1*, *ACTB*, *GAPDH*, *HMBS* and *PFKP*, was analysed by NormFinder and further cross-validated by assessing results generated following application of different combinations of potential reference genes for normalisation of the RT-qPCR data. Combining *ACTB* and *PFKP* generated high stability according to NormFinder and concordant results. Applying this normalisation, gilt derived oocytes displayed significantly higher abundance than oocytes from sows of almost all the epigenetic-related transcripts studied (*HDAC2*, *SIRT1*, *SALL4*, *KDM1A*, *KDM1B*, *KDM5A*), both before and after maturation.

**Conclusions:**

This study identified the combined use of *ACTB* and *PFKP* as the optimal normalisation for porcine oocyte RT-qPCR data. In oocytes collected from prepubertal gilts, transcription did not appear to be silenced at the time of aspiration, and accumulation of transcripts supporting histone modification facilitating proper fertilization and further embryo development seemed delayed. The results imply the epigenetic-related transcripts may have potential as markers of oocyte quality.

## Introduction

Molecules required for oocyte nuclear maturation, fertilization and early embryo development must be accumulated during the oocyte growth phase [1, 2]. In porcine oocytes, the growth phase lasts until the oocyte is enclosed in a follicle of approximately 2–3 mm in diameter, at which point transcription in the germinal vesicle nucleus is silenced prior to meiotic resumption [3–6]. Transcript abundance will subsequently gradually decline, as the oocyte relies on post-transcriptional regulation for selective degradation and usage until embryonic genome activation (EGA) [4, 5, 7, 8], which occurs around the 4-cell stage in porcine embryos [9, 10]. Gene expression in oocytes therefore represents a peculiar case where it does not merely reflect the cell's current requirement for protein synthesis, but also the cellular stage of development.

Three types of oocyte maturation can be considered; cytoplasmic, nuclear, and epigenetic [11], all of which are essential for oocytes to acquire developmental competence. Oocyte epigenetic maturation is required for establishment of maternal imprinting [2, 12], regulation of transcription [4, 13], chromosome segregation [14, 15] and to support EGA [9, 12, 16]. These are processes which take place at different times during oocyte and early embryo development.

In reverse transcription quantitative polymerase chain reaction (RT-qPCR) studies, appropriate normalisation of mRNA abundance is a crucial step for generating reliable results [17–19]. It is imperative to validate reference genes for each new oocyte maturation study, as their stability varies between species [7, 20], and is even affected by different RNA extraction [18] and cDNA synthesis protocols [7]. Most studies use endogenous reference genes, applying the recommendation of combining at least two validated reference genes of independent cellular function to avoid co-regulation [17, 19, 21]. Different algorithms have been developed for evaluating the stability of reference genes, e.g., GeNorm [22] and NormFinder [23]. Identifying optimal reference genes can, however, be challenging in oocyte and early embryo developmental studies, as the stage of the developmental process can be expected to also affect reference gene expression [19, 20, 24]. Besides stability, it is rarely discussed how to evaluate different expression profiles between reference genes, which could potentially and critically affect the results obtained after normalisation.

The first aim of this study was to validate the use of reference genes for oocyte in vitro maturation gene expression studies. The second aim was to examine the abundance of transcripts supporting histone modification during oocyte and early embryo development in a model of oocytes of low and high developmental competence. The final aim was to assess the potential of these epigenetic related genes as markers of oocyte quality. This was performed by analysing gene expression in immature and in vitro matured oocytes collected from prepubertal gilts and sows.

## Materials and methods

### Chemicals and media

All chemicals and reagents were purchased from Sigma-Aldrich (Oslo, Norway) unless otherwise stated. Porcine X medium (PXM) was used for washing cumulus-oocyte complexes (COCs) and porcine oocyte medium (POM) for oocyte maturation [25] with minor modifications to the POM (20 ml/l BME amino acids, 10.0 ml/l MEM non-essential amino acids, 5.0 mM glucose, 0.2 mM Na-pyruvate, 0.6 mM L-cysteine, 2.0 mM Ca-(lactate)_2_·5H_2_O, 2.0 mM l-glutamine, 108 mM NaCl, 10 mM KCl, 25 mM NaHCO_3_, 0.4 mM MgSO_4_·7H_2_O, 0.35 mM KH_2_PO_4_, 5.0 mM hypotaurine, 50 µM β-mercaptoethanol (Gibco, Fisher Scientific AS, Oslo, Norway), 10 ng/ml epidermal growth factor, 0.01 mg/ml gentamicin, FLI (FGF2 40 ng/mL, LIF 20 ng/mL, IGF1 20 ng/mL) and 4.0 mg/ml BSA).

### Animal material and ethics

Gilt and sow ovaries, from random herds, were collected from May to August 2022 at a commercial abattoir. No ethical approval was required as material was collected from animals that were routinely slaughtered. In Norway, swine are reared according to internationally recognized regulations and guidelines (The Animal Welfare Act, 10 July 2009, https://www.regjeringen.no/en/dokumenter/animal-welfare-act/id571188/ and Regulations for keeping pigs in Norway, 18 February 2003, https://lovdata.no/dokument/LTI/forskrift/2003-02-18-175).

### Experimental design

As a model for oocytes of low and high developmental competence [8, 26–29], immature COCs were collected from prepubertal gilts and sows. Ovaries were selected as described in Haug et al. [30]. The phrasing prepubertal gilts refers to young pigs that have not yet had a litter. In this study these were around 5 months old. Their ovaries were characterised by smaller follicles and no signs of ovulation (no corpora albicantia, corpora lutea or preovulatory follicles), in contrast to ovaries from cycling gilts which have follicles of different sizes in addition to corpora lutea or corpora albicantia. Sows are older pigs which have produced at least a first litter. Sow ovaries with follicles as well as corpora lutea or corpora albicantia from previous cycles were harvested. At the abattoir, prepubertal and cycling gilts were slaughtered separately from sows, and ovaries from prepubertal gilts and sows were collected and kept apart. COCs from both prepubertal gilts and sows were randomly allocated to the immature or in vitro maturation groups, where RNA extraction from oocytes was conducted either directly after aspiration or following 44 h in vitro maturation, respectively. Four groups were analysed: Immature oocytes from prepubertal gilts and adult sows and in vitro matured oocytes from prepubertal gilts and adult sows. In all groups, RNA extraction and downstream analyses were carried out from three biological replicates of 50–70 oocytes.

### Oocyte collection and in vitro maturation

Ovaries from sows and prepubertal gilts, in different phases of the oestrus cycle, were collected immediately after slaughter and conveyed to the laboratory in 0.9% NaCl keeping 32–36 °C within 2 h of slaughter. Subsequently, ovaries were washed in 0.9% NaCl supplemented with 2.5 µg/ml kanamycin, transferred to a beaker containing the same solution and incubated at 34–35 °C until follicle aspiration. Using an 18-gauge needle and 10 ml syringe, COCs from 2 to 6 mm diameter follicles were aspirated. Selection of oocytes were done by microscopy, including oocytes with evenly granulated cytoplasm and compact cumulus, followed by washing through three drops of PXM and one drop of POM medium. For in vitro maturation, COCs were cultured in groups of 25–30 in Nunc® four-well dishes containing 500 µl of pre-equilibrated POM medium. The first 20 h, COCs were incubated in POM supplemented with 0.1 mM dibutyryl-cAMP (dbcAMP) and 0.05 IU/ml porcine follicle-stimulating hormone (FSH) and luteinizing hormone (LH) (Insight Biotechnology Ltd, Wembley, UK). The following 24 h COCs were matured in POM without dbcAMP and the hormones FSH and LH. COCs were cultured for a total of 44 h at 38.8 °C in an humified atmosphere of 6% CO_2_ in air.

### RNA extraction and cDNA synthesis

Oocytes and cumulus cells were separated by 1 min vortexing, followed by pipetting to remove any remaining cumulus cells. Oocytes with cumulus cells still attached after this procedure were discarded. Oocytes were then washed through 6 drops of PXM, followed by 3 drops of diethyl pyrocarbonate (DEPC)-treated phosphate buffered saline (PBS) supplemented with 0.1% polyvinyl alcohol (PVA) (dPBS/PVA). The oocytes were added to the RNA extraction solution within 2 µl dPBS/PVA. Total RNA was extracted by RNAGEM™ Tissue PLUS (ZyGEM, Hamilton, New Zealand) and subsequently treated with DNase I (at 80 units/mL for 10 min, a small adjustment from the manufacturer’s guidelines). In all samples, RNA concentrations were measured employing a Qubit™ fluorometer and Qubit™ RNA High Sensitivity assay kit (Invitrogen, Oslo, Norway). Subsequently, RNA samples were stored at − 80 °C. Equal amounts of RNA, 70 ng, from each pool of oocytes were reverse transcribed into cDNA employing SuperScript IV VILO Master Mix (Invitrogen, Oslo, Norway), according to manufacturer's directions. The cDNA samples were diluted 1:20 with PCR-grade water and frozen at − 20 °C. From each preparation, one RNA sample was processed without reverse transcriptase (no-RT) to serve as a negative control in gene expression analyses.

### Quantitative PCR

Gene expression was evaluated by quantitative polymerase chain reaction (qPCR) of cDNA samples applying TaqMan® Gene Expression FAM-labelled Assays (Applied Biosystems, Foster City, California) accessible on the ThermoFisher website (http://www.thermofisher.com). Specifics of individual genes are found in Table 1 and Appendix Table 3. Each qPCR reaction mix consisted of 5 μl of (2X) TaqMan Fast Advanced Master Mix (Applied Biosystems catalogue no. 4444556), 0.5 μl (20X) TaqMan Assay (containing the primers and probes), 4.0 μl cDNA template diluted 1:20 and 0.5 μl dH_2_O, giving a total reaction volume of 10 μl. Three technical replicates were performed for each PCR reaction. QPCR was performed employing a 7500 Fast Real-Time PCR System (Applied Biosystems) in fast cycling mode. The qPCR reaction mixture underwent an initial enzyme activation at 95 °C for 20 s, followed by 45 cycles of denaturation at 95 °C for 3 s and annealing and elongation at 60 °C for 30 s. Negative controls were always included, both PCR reactions consisting of qPCR reaction mixture with no added sample cDNA template to ensure absence of nucleic acid contamination and no-RT “cDNA” samples to check for genomic DNA contamination. All no-template controls and no-RT-controls yielded no detectable amplification.

**Table 1:** Summary of genes analysed by reverse transcription quantitative PCR in porcine oocytes

Gene symbol	Full name	Accession number	TaqMan® Assay
*ACTB*	Actin beta	XM_003124280.5^a^	Ss03376563_uH
*GAPDH*	Glyceraldehyde-3-phosphate dehydrogenase	NM_001206359.1^a^	Ss03375629_u1
*HMBS*	Hydroxymethylbilane synthase	NM_001097412.1^a^	Ss03388782_g1
*HPRT1*	Hypoxanthine phosphoribosyltransferase 1	NM_001032376.2^a^	Ss03388274_m1
*YWHAG*	Tyrosine 3-monooxygenase/tryptophan 5-monooxygenase activation protein gamma	XM_005661962.3	Ss06938931_s1
*PFKP*	Phosphofructokinase, platelet	XM_021065066.1	Ss06887532_m1
*ALDOA*	Aldolase, fructose-bisphosphate A	XM_021087995.1^a^	Ss06920688_m1
*G6PD*	Glucose-6-phosphate dehydrogenase	XM_003360515.5^a^	Ss02690824_g1
*TMLHE*	Trimethyllysine Hydroxylase, Epsilon	XM_003135511.4^a^	Ss06886117_m1
*POU5F1*	POU class 5 homeobox 1	NM_001113060^a^	Ss03389800_m1
*HDAC2*	Histone deacetylase 2	XM_001925318.6	Ss02809699_m1
*KDM1A*	Lysine demethylase 1A	NM_001112687.1^a^	Ss03389746_m1
*KDM1B*	Lysine demethylase 1B	XM_001927844.5^a^	Ss06865644_g1
*KDM5A*	Lysine demethylase 5A	XM_021092486.1	Ss06875111_m1
*SALL4*	Spalt-like transcription factor 4	NM_001114673.1^a^	Ss03390075_m1
*SIRT1*	Sirtuin1	NM_001145750.2^a^	Ss03374091_m1

^a^TaqMan® Assay targets specific transcript variants, see Appendix Table 3 for details

The expression stability of five references genes, previously validated and/or utilised as reference genes in similar studies; Actin beta (*ACTB*) [18, 31–33], glyceraldehyde-3-phosphate dehydrogenase (*GAPDH*) [9, 18, 31, 34], hypoxanthine phosphoribosyltransferase 1 (*HPRT1*) [35], tyrosine 3-monooxygenase/tryptophan 5-monooxygenase activation protein gamma (*YWHAG*) [18] and hydroxymethylbilane synthase (*HMBS* aka *PBGD*) [36, 37], were compared employing the software program NormFinder [23]. GeNorm [22] is perhaps the most widely used algorithm for assessing reference gene stability. However, NormFinder has the advantage of evaluating the independent stability of individual genes by combining the inter- and intragroup variation into a stability value. NormFinder also returns the combination of two reference genes generating best stability, with better control of coregulation than GeNorm [38]. GeNorm and NormFinder have been reported to give similar results [38, 39], and both recommend stability values below 0.15, with lower values representing higher stability [22, 39]. As we were interested in individual reference gene stability, and satisfactory stability was achieved by combining two reference genes, NormFinder was applied. This study aimed for similar amounts of RNA in all samples as this is described as a prerequisite for using NormFinder and the other algorithms [40].

Cq values attained from LinRegPCR software (version 2021.2; https://medischebiologie.nl/files/) [41, 42], were applied for all calculations. Non-normalised relative abundance of each reference gene was calculated using the 2^−ΔCq^ method, with ΔCq being the Cq value of the sample minus the Cq values of the calibrator samples [7, 18]. The relative expression of each experimental gene was determined by the ΔCt method with efficiency correction according to Pfaffl [43], after normalising to the different individual reference genes. When normalising to the combination of two reference genes, the equation from Pfaffl [43] was modified according to the method described in Vandesompele et al. [22]. Amplification profiles of individual samples were utilised to calculate the mean PCR efficiency values for each primer set using LinRegPCR software.

To evaluate the relative change in gene expression before vs. after oocyte in vitro maturation, transcript abundance of in vitro matured oocytes was analysed relative to that in immature oocytes (immature as calibrator) for prepubertal gilts and sows separately. To evaluate the difference in gene expression between prepubertal gilts and sows at the same stage of maturation, transcript abundance in gilt derived oocytes was analysed relative to that of sows (sow as calibrator), both pre- and post-maturation.

### Statistical analysis

Statistical analysis was executed using RStudio version 4.1.2 (2021-11-01). Gene expression ratios were tested for normality using the Shapiro–Wilk test after log base 10 transformation. Normally distributed data was analysed by two sample *t*-test assuming unequal variance. Results were recognised as statistically significant when p ≤ 0.05. Graphs were plotted using Microsoft Excel (16.0.1).

## Results and discussion

### Validating reference genes for the porcine oocyte maturation study

When studying subtle differences in gene expression, appropriate normalisation of the RT-qPCR data is of extra importance to achieve reliable results [21, 22]. In our first analysis of five commonly used reference genes, NormFinder ranked the individual reference genes most to least stable as *ACTB*, *GAPDH*, *HPRT1*, *YWHAG* and *HMBS*. The best combination of two reference genes was *ACTB* and *HPRT1*, with a close to acceptable stability value (0.158). Excluding *HPRT1*, because of concerns regarding lack of concordance with similar studies as discussed below, NormFinder found the best combination of two reference genes, *ACTB* and *YWHAG*, to yield inferior stability compared to employing *ACTB* alone. This demonstrates that following the recommendation of combining reference genes must be accompanied by verifying stability levels.

As none of the reference genes, nor combinations thereof, exhibited optimal intra- and inter-group stability according to NormFinder, other genes were considered. Genes encoding enzymes involved in glycolysis are considered good reference gene candidates [44]. PFKP, a rate limiting enzyme for glycolysis [45], did not demonstrate any significant change in transcript abundance during porcine COC maturation in a similar study [30]. NormFinder identified *PFKP* to be the most stably expressed single gene (Table 2) and *ACTB* and *PFKP* as the best combination of two reference genes, generating an acceptable stability value of 0.149. Moreover, combining *ACTB* and *PFKP* fulfils the recommendation of the genes having separate cellular functions to avoid co-regulation [17, 19, 21].

**Table 2:** Stability values obtained from NormFinder for the analysed reference genes. Lower values representing higher stability

Gene name	Stability values
*PFKP*	0.234
*ACTB*	0.267
*GAPDH*	0.311
*HPRT1*	0.326
*YWHAG*	0.385
*HMBS*	0.452

**Fig. 1:**
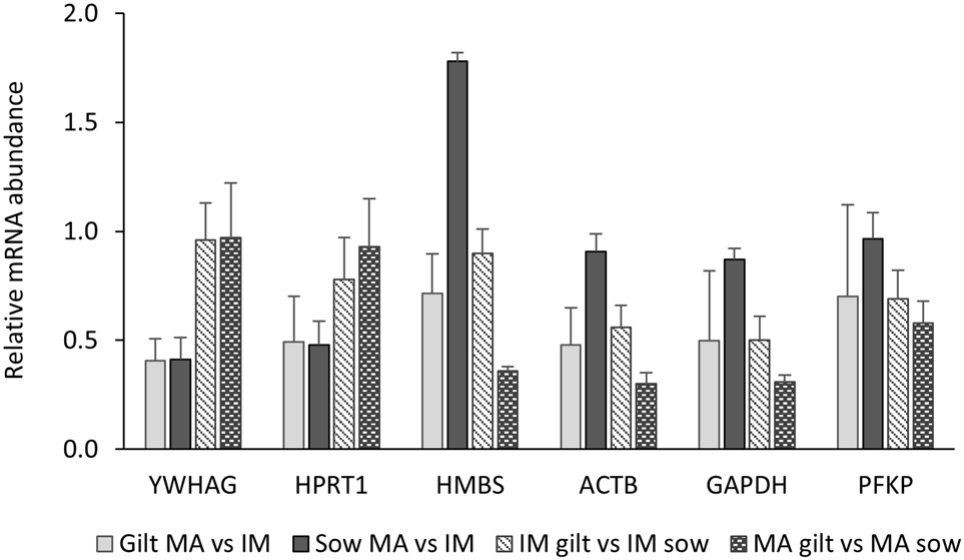
Relative transcript abundance of the potential reference genes *YWHAG, HPRT1, HMBS, ACTB*, *GAPDH* and *PFKP* in oocytes isolated from immature (IM) and in vitro matured (MA) cumulus-oocyte complexes derived from prepubertal gilts and sows as determined by reverse transcription quantitative PCR. Data are expressed as mean ± SEM of three biological replicates per group with in vitro matured transcript levels expressed relative to those in immature oocytes (Sow MA vs. IM and Gilt MA vs. IM) and gilt transcript levels expressed relative to those in sow oocytes (IM gilt vs. IM sow and MA gilt vs. MA sow). Non-normalised relative expression of each reference gene was calculated by subtracting the Cq value of the calibrator samples from the Cq value of the sample using the 2^− ΔCq^ method

Varying expression profiles between the reference genes can potentially generate different results when applied to normalise the experimental genes. Therefore, besides stability, coherence of the results, after normalising to potential reference genes, should be thoroughly considered. Relative, non-normalised transcript abundance of the different reference genes is shown in Fig. 1.

**Fig. 2:**
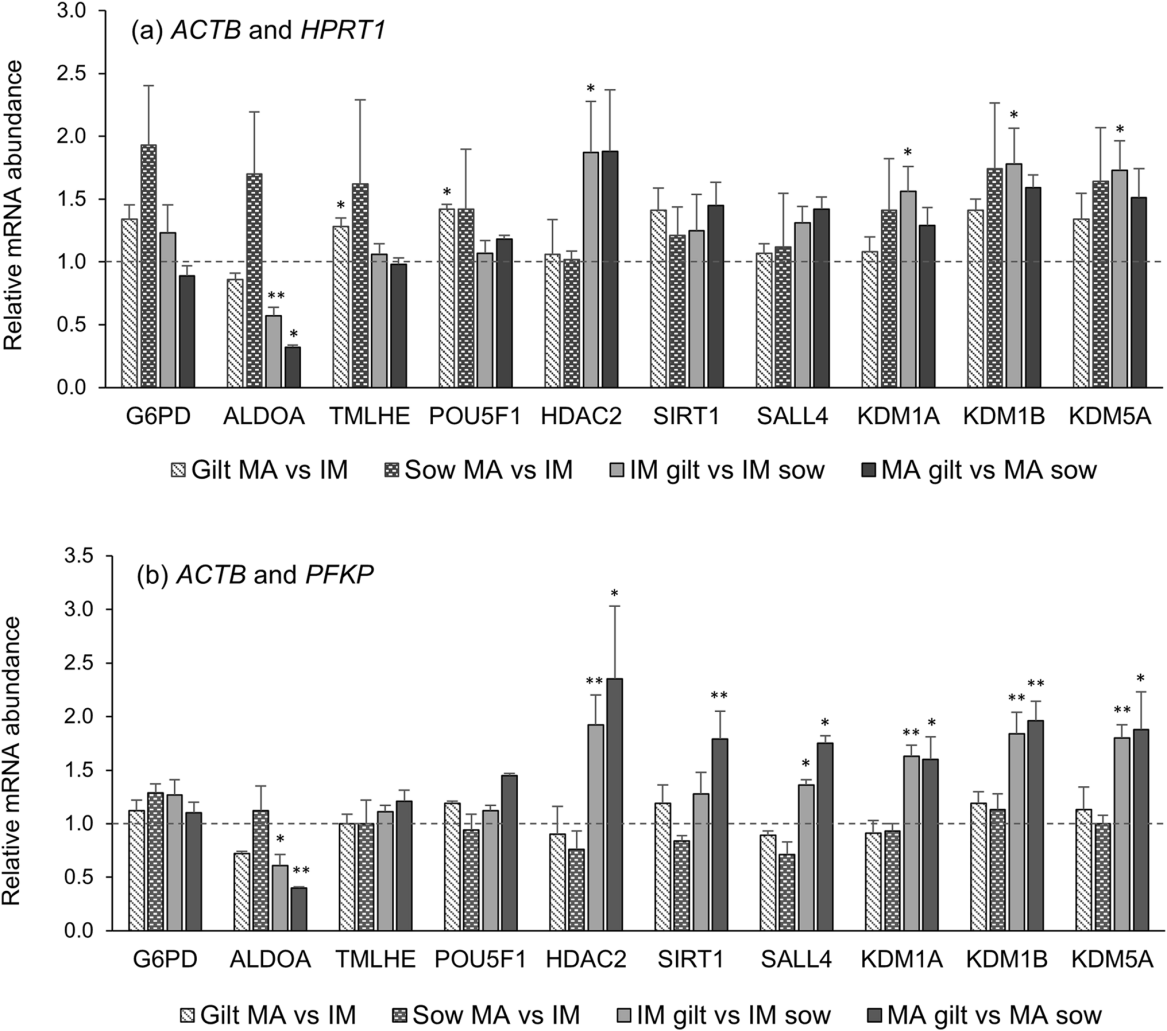
Relative transcript abundance of selected target genes in oocytes isolated from immature (IM) and in vitro matured (MA) cumulus-oocyte complexes derived from prepubertal gilts and sows as determined by reverse transcription quantitative PCR. Data are expressed as mean ± SEM of three biological replicates per group with in vitro matured transcript levels expressed relative to those in immature oocytes (Sow MA vs. IM and Gilt MA vs. IM) and gilt transcript levels expressed relative to those in sow oocytes (IM gilt vs. sow and MA gilt vs. sow). Relative expression was calculated using the ΔCq method with efficiency correction after normalising to the combination of **a** *ACTB* and *HPRT1* and **b** *ACTB* and *PFKP* according to the method described in Vandesompele et al. [22]. The dotted line represents the calibrator/control value of 1. ** indicates p < 0.05 while * indicates p < 0.10 between the compared groups

Normalising the RT-qPCR data for the epigenetic related genes (*HDAC2*, *SIRT1*, *SALL4*, *KDM1A*, *KDM1B*, *KDM5A*) by the different reference genes and most stable combinations thereof, generated different results. However, applying e.g., *ACTB* and *PFKP* combined or *ACTB* alone for normalisation generated similar patterns for all the epigenetic related genes (Fig. 2b and Appendix Fig. 3a). Therefore, a selection of genes with other functions (encoding metabolic enzymes and one transcription factor) (Table 1) were included for the purpose of validating the reference genes. These implied that the ratios obtained for the epigenetic-related genes were not a result of basal gene expression incorrectly identified by suboptimal normalisation. All the ratios obtained are given in Appendix Table 4. Concordance of the results applying the two reference gene combinations with best stability were assessed and discussed below.

Relative transcript abundance after normalising the RT-qPCR data to the combination of *ACTB* and *HPRT1*, is shown in Fig. 2a and applying *ACTB* and *PFKP* in Fig. 2b. Both combinations gave the same tendency for gene expression when comparing gilts to sows, albeit to a lesser degree with *ACTB* and *HPRT1*. However, comparing in vitro matured to immature oocytes, *ACTB* and *HPRT1* generated a different pattern than applying *ACTB* and *PFKP* for normalisation. Applying only *HPRT1* for normalisation (Appendix Fig. 3b), generated on average a more than two-fold increase in transcript abundance of mature relative to immature sow oocytes. Gilt oocytes demonstrated the same relationship but to a lesser degree. Although the results were for the most part above the significance p-value, this pattern is worth questioning, as it implies sow oocytes were less advanced than prepubertal gilt oocytes at the time of aspiration and transcriptional quiescence occurred at a later stage. This contradicts numerous previous studies of prepubertal vs. adult oocyte donors [1, 8, 26, 31, 32] and *HPRT1* should be applied with caution as a reference gene in oocyte maturation studies as it might not give results in accordance with similar studies. Interestingly, O’Connor et al. [7] reported a similar (unpublished) observation, where employing only *GAPDH* or *HPRT1* for normalisation of oocyte RT-qPCR data generated completely different expression profiles of the experimental genes. Although the influence of *HPRT1* was reduced by *ACTB*, these transcripts combined as reference still yielded questionable results.

Normalising the data using the combination of *ACTB* and *PFKP* generated similar results as when applying *ACTB* (Appendix Fig. 3a) and *PFKP* (Appendix Fig. 3c) individually. Gilt oocytes did exhibit significantly higher transcript abundance than sow oocytes for all the epigenetic related genes, a correlation which has also been observed in similar gilt and sow studies [34]. This could be explained by transcription being silenced in sow oocytes prior to follicle aspiration, with a subsequent gradual decline in transcript abundance. Gilt oocytes do not appear to have completed the accumulation of RNA and the associated transcriptional quiescence at the time of aspiration. This implies prepubertal gilt oocytes are less developed at aspiration than sow oocytes, in accordance with similar studies [27, 31, 32]. Altogether, *ACTB* and *PFKP* combined appeared to provide the most appropriate normalisation of the oocyte RT-qPCR data, by demonstrating acceptable stability and generating results consistent with expectations from the literature.

Extra caution is needed in selecting reference genes in oocyte maturation studies. NormFinder or an alternative algorithm should be applied to identify the reference gene combination with best stability, as simply combining reference genes does not necessarily lead to better stability. Thorough background knowledge of the cell types and processes being studied is essential to further critically judge the results obtained after normalising the RT-qPCR data. One should, however, take care not to discard potentially controversial results, rather recognizing that “extraordinary claims require extraordinary evidence” [46]. The RT-qPCR data for the epigenetic related genes applying *ACTB* and *PFKP* for normalisation is discussed in more detail below.

### Abundance of transcripts supporting histone modification in contrasting oocytes

Histone deacetylases (HDACs) and Sirtuins (NAD + -dependent HDACs) are involved in regulating transcription during oocyte development [4, 15]. Histone acetylation is reduced at the time of meiotic resumption [4], which is assumed to be involved in silencing transcription [3]. Histone deacetylase 2 (*HDAC2*) may be the family member having highest influence on oocyte development and maturation [15]. In addition to regulating transcription, *HDAC2* is required for deacetylation of H4K16, essential for chromosome segregation at meiotic resumption [14, 15]. Sirtuin1 (*SIRT1*) is described as a potential marker of oocyte quality [14, 47]. Besides being involved in regulating oxidative stress and metabolic processes [4, 47], SIRT1 mediates deacetylation of H3K9 which facilitates increased H3K9me3, a strong suppressive epigenetic mark [48]. Oocytes with incomplete H3K9me3 do not achieve overall transcriptional silencing and show reduced developmental potential [3]. Both *HDAC2* and *SIRT1* displayed significantly higher abundance in gilts than in sow oocytes, and more pronounced after maturation (Fig. 2b). The data indicates gilt oocytes have not completed the accumulation of these transcripts and transcription is not silenced at the time of aspiration, and possibly not prior to meiotic resumption. In future studies, it would be beneficial to also analyse gene expression after 20 h of maturation, at which point meiotic resumption is induced, to assess transcriptional activity both before and after this time point. The delayed accumulation of *HDAC2* could potentially affect chromosome segregation at meiotic resumption.

Demethylation of H3K4me3 by KDM5 proteins is crucial for proper EGA [9, 12, 16]. Of the KDM5 family, only lysine demethylase 5A (*KDM5A*) transcripts were detected in porcine oocytes [9]. These transcripts are stored during the oocyte growth phase [1, 2]. If the oocyte had completed the growth phase and transcription was globally silenced at the time of aspiration it would be expected to observe little alteration in *KDM5A* transcript abundance during maturation, as observed for sows in Fig. 2b. The transcript abundance of *HDAC2* and *KDM5A*, acting at different stages of oocyte and early embryo development, could aid in reference transcript validation.

The other epigenetic related genes all displayed similar patterns of higher abundance in gilt than sow oocytes. This was lysine demethylase 1A (*KDM1A)*, important in the maintenance of meiotic arrest and demethylation of H3K4me1 and H3K4me2 prior to EGA [12], lysine demethylase 1B (*KDM1B*), required for the establishment of maternal genomic imprints during oocyte growth [2, 12] and Spalt-like transcription factor 4 (*SALL4*), regulating e.g. transcription of several histone demethylases and described as pivotal for oocyte maturation and resumption of meiosis [11].

This study found that oocytes derived from prepubertal gilts appeared to be delayed in the accumulation of transcripts supporting histone modifications facilitating maternal imprinting, silencing of transcription, meiotic resumption and EGA. This is in accordance with Braga et al. [31] and Masala et al. [1], who investigated processes involved in DNA-methylation, applying the same model of prepubertal vs. adult oocyte donors in pig and sheep, respectively. They concluded, as in agreement with the present study, oocytes collected from premature animals had not completed accumulation of mRNA prior to in vitro maturation [1, 31] nor DNA methylation reprogramming at the end of maturation [31]. Oocytes collected from prepubertal gilts indicated delayed metabolism compared to oocytes from sows [30], which could, in addition to the above-mentioned factors, influence the establishment of epigenetic modifications that require the availability of different metabolic intermediates [49, 50]. The delayed accumulation of transcripts involved in epigenetic reprogramming in oocytes collected from premature animals could lead to failed fertilization, aneuploidy and arrested or abnormal embryonic development [3, 4]. Lower abundance of these gene transcripts post maturation could possibly predict oocytes of higher quality. However, in oocyte maturation studies, the timing of transcriptional silencing affects transcript abundance and the interpretation thereof. Further studies would be required to validate the lower abundance of these transcripts as a quality parameter, and whether it could be generalised beyond the gilt/sow oocyte model.

## Conclusions

This study identified *PFKP*, used in combination with *ACTB*, to generate the most appropriate normalisation for the porcine oocyte maturation gene expression data. When comparing prepubertal gilt to sow oocytes, there has been a lack of information regarding transcripts supporting histone modification during oocyte and early embryo development. The results of this study indicated transcription was not silenced in gilt oocytes at the time of aspiration, possibly not prior to meiotic resumption, and accumulation appeared to be delayed of transcripts supporting histone modifications involved in maternal imprinting, meiotic resumption and EGA. This may perturb fertilization and further embryo development. Further investigations are required to establish the potential use of these transcripts as oocyte quality parameters.

## Figures and Tables

**Fig. 3:**
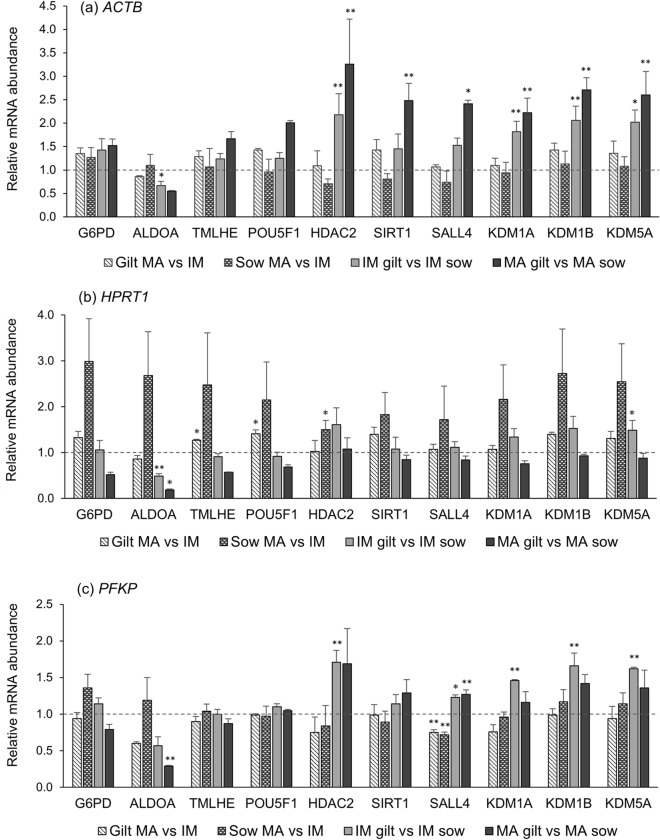
Relative transcript abundance of selected target genes in oocytes isolated from immature (IM) and in vitro matured (MA) cumulus-oocyte complexes derived from prepubertal gilts and sows as determined by reverse transcription quantitative PCR. Data are expressed as mean ± SEM of three biological replicates per group with in vitro matured transcript levels expressed relative to those in immature oocytes (Sow MA vs. IM and Gilt MA vs. IM) and gilt transcript levels expressed relative to those in sow oocytes (IM gilt vs. sow and MA gilt vs. sow). Relative expression was calculated using the ΔCq method with efficiency correction [43], after normalising to **a** *ACTB*, **b** *HPRT1* and **c** *PFKP*. The dotted line represents the calibrator value of 1. **indicates p < 0.05 while *indicates p < 0.10 between the compared groups

## Data Availability

The data supporting the findings of this study are available from the corresponding author upon reasonable request.

## Appendix

See Tables

**Table 3:** Complete list where the TaqMan® Assay will detect specific transcript variants

Gene symbol	TaqMan® Assay	Accession numbers of transcript variants
*ACTB*	Ss03376563_uH	XM_003124280.5 (X1), XM_021086047.1 (X2)
*GAPDH*	Ss03375629_u1	XM_021091114.1 (X2)
*HMBS*	Ss03388782_g1	XM_021102144.1 (X1), XM_021102146.1 (X2), XM_021102147.1 (X3), XM_021102148.1 (X4)
*HPRT1*	Ss03388274_m1	XM_021079503.1 (X1), XM_021079504.1 (X2)
*ALDOA*	Ss06920688_m1	XM_021087995.1 (X1), XM_021087996.1 (X2), XM_021087997.1 (X3), XM_021087998.1 (X4), XM_021088000.1 (X6), XM_021088001.1 (X7), XM_021088002.1 (X8), XM_021088004.1 (X9)
*G6PD*	Ss02690824_g1	XM_021080744.1 (X1), XM_003360515.5 (X2)
*TMLHE*	Ss06886117_m1	XM_003135511.4 (X1), XM_021080447.1 (X2), XM_021080448.1 (X3), XM_021080450.1 (X4), XM_005674022.3 (X5)
*POU5F1*	Ss03389800_m1	XM_021097869 (X1)
*KDM1A*	Ss03389746_m1	XM_021093198.1 (X1), XM_021093199.1 (X2), XM_021093200.1 (X3)
*KDM1B*	Ss06865644_g1	XM_001927844.5 (X1), XM_013977460.2 (X2)
*SALL4*	Ss03390075_m1	XM_021077202.1 (X1)
*SIRT1*	Ss03374091_m1	XM_021072203.1 (X1), XM_021072204.1 (X2)

3,

**Table 4:** Relative transcript abundance acquired for ten genes of interest in oocytes isolated from immature and in vitro matured cumulus-oocyte complexes derived from prepubertal gilts and sows as determined by reverse transcription quantitative PCR

	*G6PD*	*ALDOA*	*TMLHE*	*POU5F1*	*HDAC2*	*SIRT1*	*SALL4*	*KDM1A*	*KDM1B*	*KDM5A*
Mature sow vs. immature sow oocytes:										
*YWHAG*	2.96	2.55	2.58	2.25	1.58	1.87	1.77	1.16	2.68	2.52
*HPRT1*	2.99	2.68	2.48	2.15	1.50	1.80	1.72	2.16	2.73	2.55
*HMBS*	0.66	0.55	0.57	0.51	0.39	0.43	0.39	0.49	0.58	0.55
*ACTB*	1.27	1.10	1.07	0.96	0.71	0.81	0.74	0.94	1.13	1.08
*GAPDH*	1.40	1.20	1.10	1.03	0.88	0.93	0.76	1.01	1.20	1.17
*PFKP*	1.36	1.19	1.04	0.97	0.84	0.89	0.72	0.96	1.17	1.14
*ACTB* + *HPRT1*	1.93	1.70	1.62	1.42	1.02	1.21	1.12	1.41	1.74	1.64
*ACTB* + *PFKP*	1.29	1.12	1.03	0.94	0.76	0.84	0.71	0.93	1.13	1.09
Mature gilt vs. immature gilt oocytes:										
*YWHAG*	1.76	1.13	1.65	1.85	1.32	1.84	1.40	0.86	1.81	1.69
*HPRT1*	1.33	0.86	1.27	1.41	1.00	1.40	1.07	1.07	1.40	1.31
*HMBS*	0.93	0.58	0.91	0.98	0.81	1.01	0.72	0.78	1.00	0.98
*ACTB*	1.35	0.86	1.29	1.43	1.09	1.43	1.07	1.10	1.43	1.36
*GAPDH*	1.29	0.83	1.26	1.38	1.08	1.37	1.03	1.08	1.40	1.33
*PFKP*	0.94	0.60	0.90	0.99	0.75	0.99	0.75	0.76	0.99	0.94
*ACTB* + *HPRT1*	1.32	0.86	1.28	1.42	1.06	1.41	1.07	1.08	1.41	1.34
*ACTB* + *PFKP*	1.12	0.72	1.08	1.19	0.90	1.19	0.89	0.91	1.19	1.13
Immature gilt vs. immature sow oocytes:										
*YWHAG*	0.83	0.41	0.73	0.74	1.26	0.84	0.90	0.78	1.22	1.18
*HPRT1*	1.06	0.49	0.90	0.92	1.60	1.00	1.12	1.34	1.53	1.49
*HMBS*	0.89	0.43	0.80	0.80	1.36	0.90	0.97	1.16	1.27	1.28
*ACTB*	1.43	0.67	1.24	1.25	2.18	1.45	1.53	1.82	2.06	2.02
*GAPDH*	1.61	0.76	1.39	1.41	2.45	1.64	1.72	2.05	2.34	2.28
*PFKP*	1.14	0.57	1.00	1.01	1.71	1.14	1.23	1.46	1.66	1.62
*ACTB* + *HPRT1*	1.23	0.57	1.06	1.07	1.87	1.25	1.31	1.56	1.78	1.73
*ACTB* + *PFKP*	1.27	0.61	1.11	1.12	1.92	1.28	1.36	1.63	1.84	1.80
Mature gilt vs. mature sow oocytes:										
*YWHAG*	0.53	0.19	0.57	0.70	1.06	0.86	0.85	0.58	0.92	0.87
*HPRT1*	0.52	0.19	0.57	0.69	1.00	0.85	0.84	0.76	0.90	0.88
*HMBS*	1.25	0.45	1.40	1.65	2.91	2.10	1.95	1.89	2.28	2.25
*ACTB*	1.52	0.55	1.67	2.01	3.26	2.48	2.41	2.22	2.71	2.60
*GAPDH*	1.46	0.53	1.63	1.94	3.25	2.40	2.33	2.20	2.66	2.57
*PFKP*	0.79	0.29	0.87	1.05	1.69	1.29	1.27	1.16	1.42	1.36
*ACTB* + *HPRT1*	0.89	0.32	0.98	1.18	1.88	1.45	1.42	1.29	1.59	1.51
*ACTB* + *PFKP*	1.10	0.40	1.21	1.45	2.35	1.79	1.75	1.60	1.96	1.88

Data are expressed as the mean of three biological replicates per group after normalising to the reference genes *YWHAG, HPRT1, HMBS, ACTB, GAPDH* and *PFKP* and the two combinations with best stability

Transcript levels of in vitro matured oocytes are expressed relative to those in immature oocytes, and gilt transcript levels are expressed relative to those in sow oocytes

Relative expression of each reference gene was calculated using the ΔCq method with efficiency correction [43]

When normalising to the combination of two reference genes, the equation from Pfaffl [43] was modified according to the method described in Vandesompele et al. [22]

4 and Fig. 3.

## Abbreviations

EGA: Embryonic genome activation
RT-qPCR: Reverse transcription quantitative polymerase chain reaction
COC: Cumulus-oocyte complexes
FSH: Follicle-stimulating hormone
LH: Luteinizing hormone
no-RT: Without reverse transcriptase
*ACTB*: Actin beta
*GAPDH*: Glyceraldehyde-3-phosphate dehydrogenase
*HMBS*: Hydroxymethylbilane synthase
*HPRT1*: Hypoxanthine phosphoribosyltransferase 1
*YWHAG*: Tyrosine 3-monooxygenase/tryptophan 5-monooxygenase activation protein gamma
*PFKP*: Phosphofructokinase, platelet
*ALDOA*: Aldolase, fructose-bisphosphate A
*G6PD*: Glucose-6-phosphate dehydrogenase
*TMLHE*: Trimethyllysine hydroxylase, epsilon
*POU5F1*: POU class 5 homeobox 1
*HDAC2*: Histone deacetylase 2
*KDM1A*: Lysine demethylase 1A
*KDM1B*: Lysine demethylase 1B
*KDM5A*: Lysine demethylase 5A
*SALL4*: Spalt-like transcription factor 4
*SIRT1*: Sirtuin1
